# Ameloblastomas: Clinicopathological features from 70 cases diagnosed in a single Oral Pathology service in an 8-year period

**DOI:** 10.4317/medoral.19802

**Published:** 2014-08-17

**Authors:** Andressa-Incerte Filizzola, Teresa-Cristina-Ribeiro Bartholomeu-dos-Santos, Fábio-Ramôa Pires

**Affiliations:** 1 Undergraduate student, School of Dentistry, State University of Rio de Janeiro, Rio de Janeiro/RJ, Brasil; 2 DDS, MSc, Professor, Oral Pathology, School of Dentistry, State University of Rio de Janeiro, Rio de Janeiro/RJ, Brasil; 3 DDS, PhD, Professor, Oral Pathology, School of Dentistry, State University of Rio de Janeiro, Rio de Janeiro/RJ, Brasil

## Abstract

Ameloblastomas are odontogenic tumors that can present some distinct clinicopathological profiles when comparing different populations and studies. Objectives: The aim of the present study was to analyze the clinicopathological features from a series of ameloblastomas diagnosed in a single Oral Pathology service in Brazil in an 8-year period. Study Design: The files were revised and all cases diagnosed as ameloblastomas in the period were retrieved. All hematoxylin and eosin stained histological slides were reviewed and all clinical and radiological information were obtained through a review of the laboratory forms. Data were descriptively analyzed and a comparison was performed with the different ameloblastomas subtypes. Results: Seventy ameloblastomas composed the final sample, including 57 (81%) solid/multicystic, 9 (13%) unicystic, 2 (3%) desmoplastic and 2 (3%) peripheral ameloblastomas. Mean age of the affected patients was in the forth decade of life and there was a slight male predominance. Most tumors presented as multilocular radiolucencies, were located in the posterior mandible and showed the follicular and plexiform histological patterns. There was no difference on the mean age of the patients affected by solid and unicystic ameloblastomas. Conclusions: The present results showed that the clinicopathological features of the ameloblastomas included in this Brazilian sample were similar to the features described in most other worldwide populations.

** Key words:**Ameloblastoma, solid, unicystic, review, epidemiology, histology.

## Introduction

Ameloblastomas are considered one of the three most common odontogenic tumors (OT), together with odontomas and keratocystic odontogenic tumors (1,2). Their main clinical, radiological and histological characteristics have been described in the literature, but little is known about their specific geographical, ethnic and regional variations when different populations are compared. Although there are some studies focusing on the clinicopathological features from ameloblastomas in selected populations, few derived from Latin America ( Table 1) (3-14). The aim of the present study was to analyze the clinicopathological features from a series of ameloblastomas diagnosed in a single Oral Pathology service in southeastern Brazil in a 8-year period.

**Table 1:**
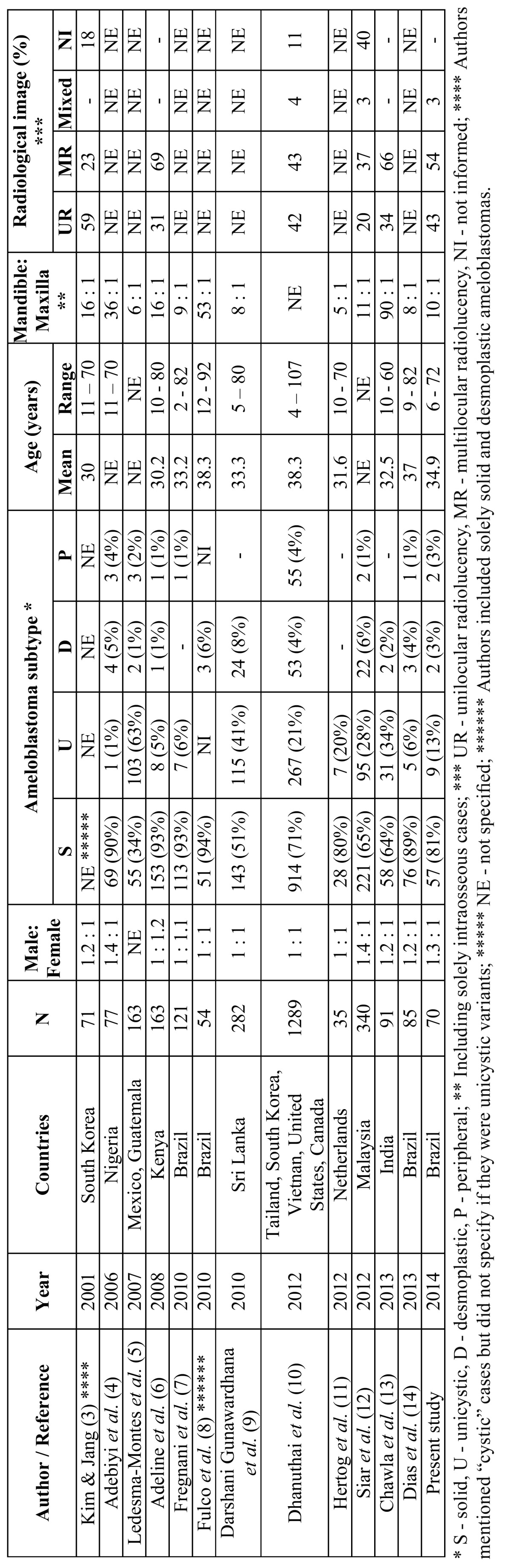
Distribution of the clinicopathological data from ameloblastomas derived from studies reported in the English-language literature from 2001 to present.

## Material and Methods

All cases diagnosed as ameloblastomas in the Oral Pathology service, School of Dentistry, State University of Rio de Janeiro, Brazil, from 2005 to 2012, were initially selected. Hematoxylin and eosin (HE) stained histological slides were reviewed in light microscopy and all cases presenting sufficient material for adequate final diagnosis and analysis of the histological variants were retrieved. Laboratory forms containing clinical and radiological information were reviewed and those containing most required information were selected to compose the final sample. Information about gender and age of the patients, anatomical location of the tumors, presence of symptoms, radiological size of the tumors (greatest diameter in milimeters), type of image (radiolucency or mixed image; unilocular or multilocular), presence of root resorption on the teeth adjacent to the tumors, radiological limits of the tumor (well-defined or ill-defined) and final histological subtype were obtained. The analysis of the radiological images was based on panoramic radiographs complemented by periapical radiographs when necessary.

HE-stained histological slides from the final sample were carefully reviewed and all cases were classified according to well-established criteria (1,2) in 4 types of ameloblastomas: solid (multicystic), unicystic, desmoplastic and peripheral. Solid and peripheral ameloblastomas were also subtyped according to the histological pattern in follicular, plexiform, acanthomatous, granular cell, basaloid and angiomatous (1). Cases presenting more than one histological subtype were classified taken in account all histological patterns observed. Unicystic ameloblastomas were additionally classified in luminal, intraluminal and mural patterns of growing.

All clinical, radiological and histological data were included in a .sav file designed specifically for the study and analyzed with the use of the Statistical Program for Social Sciences (SPSS, version 17, Chicago, IL, United States). The differences were considered statistically 

significant when *p*< 0.05 (5%). This project was approved by the Ethics in Research Committee from the State University of Rio de Janeiro (173.216/2012).

## Results

In the 8-year period from 2005 to 2012, ameloblastomas represented 1.5% of all cases and 24% of all OT diagnosed in the service. From the 70 selected ameloblastomas, 57 (81%) were diagnosed as solid/multicystic, 9 (13%) unicystic, 2 (3%) desmoplastic and 2 (3%) peripheral. Forty patients (57%) were males and 30 (43%) females and there was no statistically significant difference between the percent of males and females affected by each subtype ( Table 2). The mean age of all patients was 34.9 years (ranging from 6 to 72 years) and there was no statistically significant difference on the man age of males (36.8 years) and females (32.3 years) (*p*=0.235). There was also no statistically significant difference on the mean age of patients with solid (34.7 years) and unicystic (28.5 years) ameloblastomas (*p*=0.266). Mean age of the patients affected by both peripheral (57.5 years) and desmoplastic (43 years) ameloblastomas was higher than mean age of the patients affected by the other two subtypes.

**Table 2:**
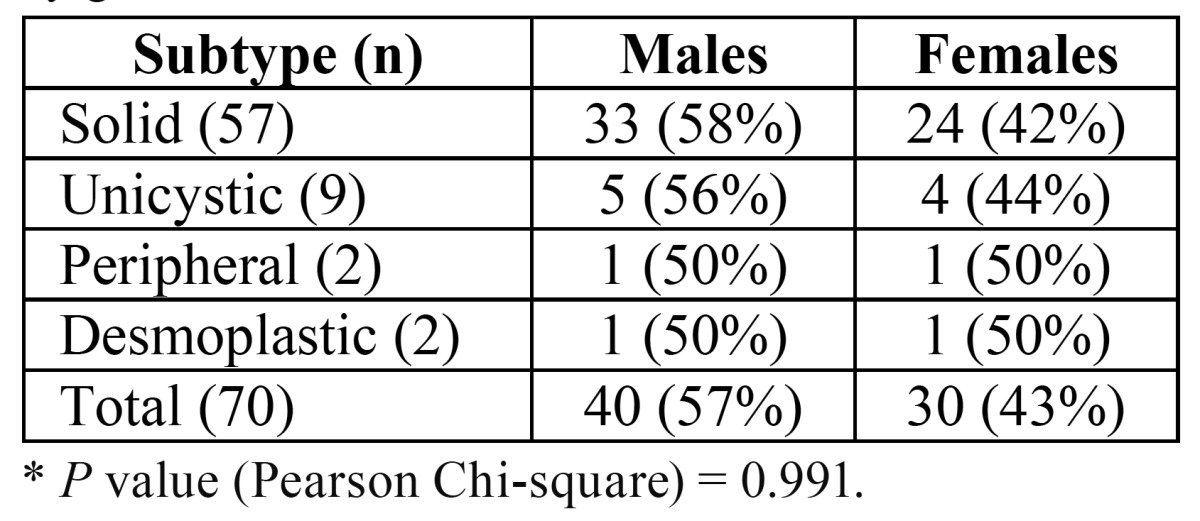
Distribution of the ameloblastoma subtypes by gender. *

One third of the patients reported symptoms associated with the tumors, including especially pain and discharge. Mandible was affected in 88% of the cases, maxilla in 9% and alveolar mucosa in 3%; the posterior region of the mandible was affected in 44 cases (64%), in contrast with the anterior region (8 cases, 11%).  Table 3 shows the anatomical distribution of the tumors by subtype. Multilocular radiolucencies characterized 51% of the cases, 41% were unilocular radiolucencies, 4% showed mixed images and 4% did not show radiological images (peripheral ameloblastomas). Radiological limits were considered well defined and ill-defined in, respectively, 80% and 20% of the cases. Root resorption was encountered in 56% of the tumors located in close proximity with the adjacent teeth. The greatest radiological diameter of the tumors ranged from 4 to 90 mm (mean of 46.2 mm).

**Table 3:**
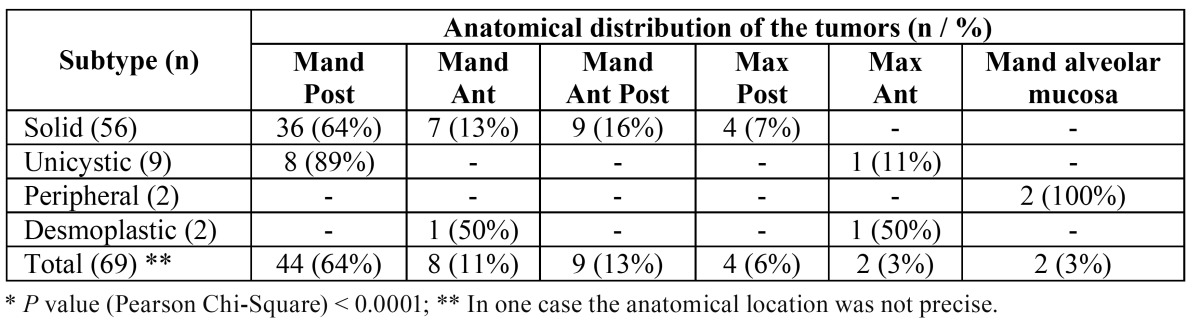
Anatomical distribution of the ameloblastoma subtypes (Mand – mandible; Max – maxilla; Post – posterior; Ant – anterior; NE – not specified). *

Histological pattern of the 57 solid ameloblastomas included: follicular (18 cases, 26%), plexiform (13, 18%), follicular + acanthomatous (10, 14%), follicular + plexiform (4, 6%), follicular + plexiform + acanthomatous (4, 6%), follicular + granular cell (3, 4%), plexiform + angiomatous (2, 3%), follicular + angiomatous (2, 3%), follicular + acanthomatous + basaloid (1, 1%). In this group of solid tumors, 42 (74%) and 23 (40%), respectively, showed the follicular and plexiform histological patterns. The 9 unicystic ameloblastomas were characterized by mural (6, 9%) and luminal (3, 4%) growing pattern and the 2 peripheral ameloblastomas were characterized by the presence of both follicular and plexiform histological subtypes.

There was a statistically significant difference on the mean age of the patients presenting symptoms or no, but there was no difference on the mean ages of patients presenting unilocular or multilocular radiolucencies and tumors presenting follicular and plexiform histological patterns ( Table 4). Mean greatest diameter of solid (47.8 mm) and unicystic (37 mm) ameloblastomas were not statistically significant different (*p*=0.306). Females and males presented tumors with mean greatest diameter of 47.6 mm and 45.2 mm, respectively (*p*=0.718). There were also no statistically significant differences on the mean greatest diameter of the tumors when comparing patients with or without symptoms, with unilocular or multilocular radiolucencies, and tumors presenting follicular or plexiform histological patterns ( Table 4). Although the plexiform histological pattern was found more frequently in males, there was no statistically significant differences on the distribution of both follicular and plexiform patterns in solid ameloblastomas from males and females ( Table 5).

**Table 4:**
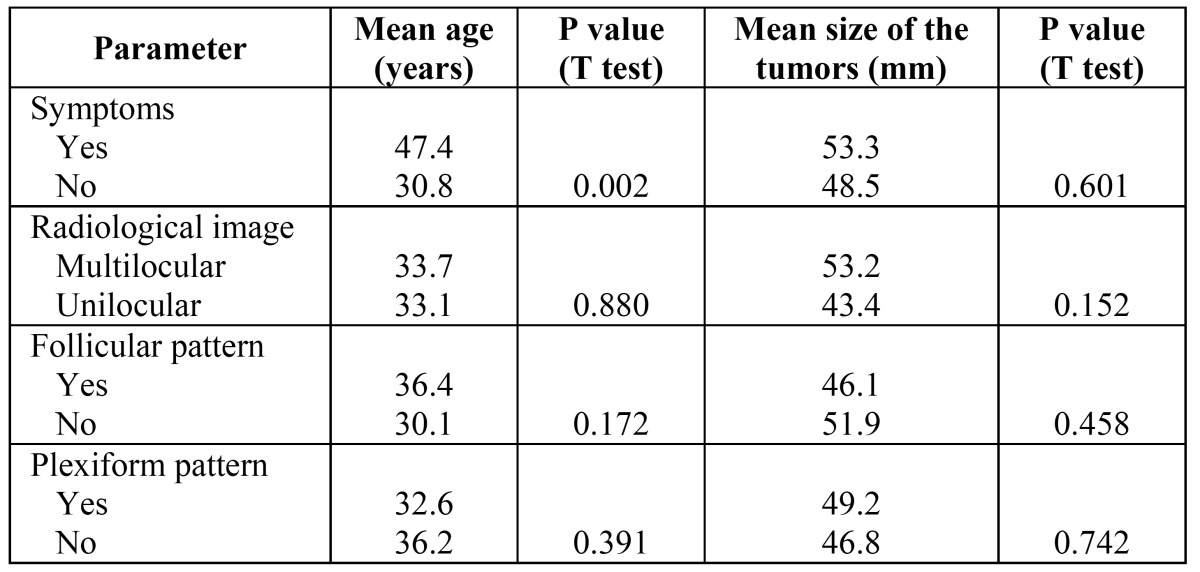
Distribution of the mean age of the patients and the mean size of the ameloblastomas according to the presence of symptoms, radiological image and presence of the follicular and plexiform histological subtype.

**Table 5:**
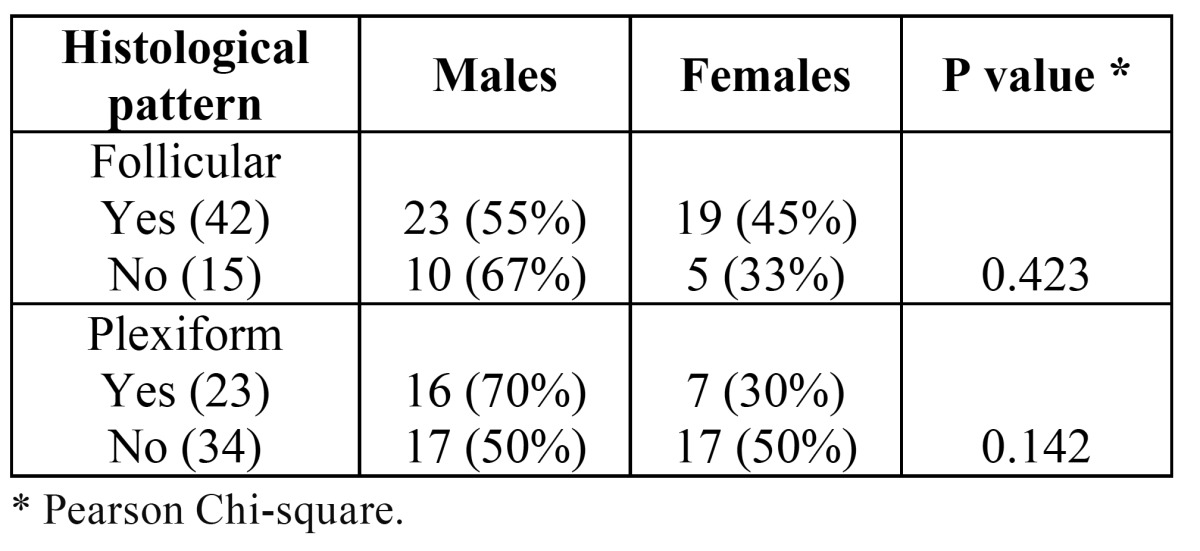
Distribution of the frequency of the follicular and plexiform histological patterns according to gender of the patients with solid ameloblastomas.

## Discussion

Ameloblastomas represent from 0.5% to 1.3% of all diagnosis (5,9,10,13) and from 22% to 46% (this higher value when excluding keratocystic odontogenic tumors) of all OT diagnosed in Oral Pathology laboratories (5,12,13). Apart from being classified as benign entities, these tumors can present local infiltrative growth and are able to produce extensive bone destruction and infiltration to the surrounding soft tissues. There are also some histologically benign ameloblastomas producing regional and distant metastasis (malignant ameloblastomas) and some malignant ameloblast-derived neoplasms (ameloblastic carcinomas) showing some histological characteristics superimposed to the ones found in ameloblastomas, bringing additional difficulties on their differential diagnosis.

Few studies have comparatively evaluated the clinical, radiological and histological features of ameloblastomas diagnosed in different populations and their results have showed some distinct profiles, suggesting the possibility of minor ethnic and geographical variations. In the last 12 years, 11 studies with similar methods than the ones used in the present one were reported in the English-language indexed literature, including North american, Latin american, African, European and Asian populations ( Table 1) (3-14). Although most information rely uniform when comparing these studies, others show distinct profiles in the included populations.

Solid ameloblastomas represent from 51% to 94% of all ameloblastomas, and are followed by unicystic ameloblastomas (1% to 41% of all ameloblastomas) ( Table 1). Ledesma-Montes *et al*. (5) have reported that 63% of their ameloblastomas derived from Mexican and Guatemalan populations were unicystic, in contrast with the results from studies including other Latin American (Brazilian) populations. Social, ethnic and geographic differences, associated with difficulties in establishing uniform diagnostic criteria and inclusion of incisional biopsy-derived together with surgical resection specimens can justify some variations in the frequency of the ameloblastomas subtypes (5,9). Peripheral and desmoplastic ameloblastomas are the less frequent subtypes and represent from 1 to 4% and from 1 to 8% of all ameloblastomas, respectively ( Table 1). The distribution of the subtypes included in the present study is in accordance with the literature.

Ameloblastomas equally affect males and females (8-10) or show a slight predilection for males (3,4,12-15), as demonstrated in the present results. Mean age of the affected patients is usually in the fourth decade of life, but these tumors can affect patients in a wide age range (3,6,7-11). There seems to exist geographical differences on the mean age of the affected patients, as Asian patients show lower mean ages than North American patients (10). Mandible is the preferred site of involvement, with a mandible:maxilla ratio varying from 5:1 to 90:1 (3-9,11-15) ( Table 1). Solid and unicystic ameloblastomas show predilection for the posterior region of the maxillary bones, as demonstrated by the present results; contrarily, desmoplastic ameloblastomas usually affect the anterior region.

Ameloblastomas can produce extensive bone destruction but are mostly characterized by well-defined radiolucencies surrounded by cortical bone sclerosis, as demonstrated by the present results. Most studies reinforce the idea that ameloblastomas are mainly characterized by multilocular radiolucencies, especially the solid subtype (6,7,12,13), but some have demonstrated an equal distribution or even predominance of unilocular radiolucencies (3,5,10). In the present study, mean greatest diameter of the tumors was 46 mm and, due to large size of some tumors, symptoms such as pain and discharge were found in one third of the cases, similarly to other studies (5-7,13). Tumors associated with symptoms affected older patients but, curiously, presence of symptoms was not directly associated with mean greatest diameter of the tumors. These features support that the presence of symptoms are not simply associated with tumor growth, but other features associated with the age of the patients, such as trauma induced by ill-fitting prosthetic appliances or advanced pulp/periodontal disease in the adjacent teeth (inducing secondary infection) would have a role. Root resorption was found in half the tumors located adjacent to remaining teeth in the present study.

Desmoplastic ameloblastomas usually affect adults in their fourth to fifth decades of life, without gender predilection, with slight predilection for the mandible and for the anterior region of the maxillary bones, manifesting as a mixed radiological image (16). Peripheral ameloblastomas are characterized by an exophytic soft-tissue gingival or alveolar mucosal mass, usually affecting adults in their fifth to sixth decades of life, with slight predilection for males and for the mandible (17). They are usually histologically characterized by the follicular and plexiform patterns, similarly to both cases included in the present study.

Unicystic ameloblastomas are neoplastic entities characterized by a cystic morphological appearance lined by an ameloblastic epithelium that can show tumoral growth to both the lumen and to the fibrous connective tissue (18). They usually affect younger patients, with mean ages in the third decade of life, with predilection for the posterior mandible (17). The results of the present study showed that, contrarily to the literature (5,6,9,10,12,13) and similarly Hertog *et al*. (11), there was no statistically significant difference in the mean age of patients affected by solid and unicystic ameloblastomas; however, the few unicystic ameloblastomas included in the sample precludes any definite conclusion. Additionally, most unicystic ameloblastomas included in the sample were classified as mural type, similarly to other studies (7,9). As this subtype shows a biological behavior and a recurrence potential similar to solid ameloblastomas, it has been considered more closely related to solid than to unicystic ameloblastomas.

Solid ameloblastomas represent the majority of ameloblastomas and are usually diagnosed in adults in their fourth decade of life, with slight predilection for males and mostly located on the posterior mandible. Histologically, solid ameloblastomas are usually characterized by the presence of both follicular and plexiform patterns and their associations and the presence of more than one histological subtype is common in an individual tumor (3-10,12,14,19), in accordance with the present results. Some studies have classified solid ameloblastomas in a specific subtype (4,14), using the predominant histological subtype as reference, but the association of histological patterns is common in these tumors, turning the evaluation of the importance of a specific pattern on ameloblastoma growth and behavior difficult. Our results did not highlight distinct patterns of age and gender predilection and size of the tumors when tumors containing the follicular and plexiform histological patterns were compared, in accordance with the literature (11).

In the present sample, solid and unicystic ameloblastomas were the two most common subtypes, mean age of the patients was on the fourth decade of life and there was a slight predilection for males. Tumors manifested predominantly as multilocular radiolucencies, mostly on the posterior mandible and the follicular and plexiform subtypes were the predominant histological patterns. There were no differences on the mean age of patients affected by solid and unicystic ameloblastomas. The profile of the tumors in the present sample was similar to the profile described in most worldwide populations.
